# Principal component analysis reveals the 1000 Genomes Project does not sufficiently cover the human genetic diversity in Asia

**DOI:** 10.3389/fgene.2013.00127

**Published:** 2013-07-04

**Authors:** Dongsheng Lu, Shuhua Xu

**Affiliations:** Max Planck Independent Research Group on Population Genomics, Chinese Academy of Sciences and Max Planck Society Partner Institute for Computational Biology, Shanghai Institutes for Biological Sciences,Chinese Academy of SciencesShanghai, China

**Keywords:** human genetic diversity, population structure, 1000 Genomes Project, Pan-Asian SNP Project, Human Genome Diversity Project, single nucleotide polymorphisms, principal component analysis

## Abstract

The 1000 Genomes Project (1KG) aims to provide a comprehensive resource on human genetic variations. With an effort of sequencing 2,500 individuals, 1KG is expected to cover the majority of the human genetic diversities worldwide. In this study, using analysis of population structure based on genome-wide single nucleotide polymorphisms (SNPs) data, we examined and evaluated the coverage of genetic diversity of 1KG samples with the available genome-wide SNP data of 3,831 individuals representing 140 population samples worldwide. We developed a method to quantitatively measure and evaluate the genetic diversity revealed by population structure analysis. Our results showed that the 1KG does not have sufficient coverage of the human genetic diversity in Asia, especially in Southeast Asia. We suggested a good coverage of Southeast Asian populations be considered in 1KG or a regional effort be initialized to provide a more comprehensive characterization of the human genetic diversity in Asia, which is important for both evolutionary and medical studies in the future.

## INTRODUCTION

Due to recent burgeoning in next generation sequencing (NGS) technologies, whole-genome sequencing of a large number of individuals in multiple populations is now feasible. This advanced technology has facilitated genome-wide investigation of human genetic variations and inference of population demographic history and natural selection without ascertainment bias (Teo et al., 2010). Pioneer efforts of 1000 Genomes Project (1KG) based on NGS at low coverage have already identified millions of variants, including single nucleotide polymorphisms (SNPs), insertion/deletions (INDELs), and structural variations (SVs) in diverse populations worldwide, which has made remarkable achievements in the studies of human genetic variation, natural selection, human disease mapping, and local adaptation to environments (Zawistowski et al., 2010; Holm et al., 2011; Pasaniuc et al., 2012). Another international effort prior to 1KG, the International HapMap Project (HapMap), investigated human common genetic variations in human populations using genotyping technology. However, both projects included only a few number of Asian population samples that were exclusively from East Asia, such as CHB (Han Chinese in Beijing, China), CHD (Chinese in Denver, CO, USA), and JPT (Japanese in Tokyo, Japan; The International HapMap Consortium, 2005), which could only represent a small proportion of human genetic diversity in Asia. The Human Genome Diversity Project (HGDP), one of the most widely used resources, also lacked good representation of samples especially from Southeast Asia. Although HGDP (Li et al., 2008) consists of many East Asian and some Oceanian populations such as Melanesian and Papuan, there is almost no Southeast Asian population sample except for a single Cambodian population. Although 1KG has newly incorporated two more Asian populations KHV (Kinh in Ho Chi Minh City, Vietnam) and CDX (Chinese Dai in Xishuangbanna) mainly from southern part of China which could be treated as Southeast Asian populations, they may only explain a very small proportion of genetic diversity in Southeast Asian populations. With the genome-wide SNP data in a large number of 47 Southeast Asian population samples investigated in the HUGO Pan-Asian SNP Project (PASNP; The HUGO Pan-Asian SNP Consortium, 2009), we have a good opportunity to examine and evaluate the coverage of human genetic diversity in 1KG data. Based on the SNP data obtained from PASNP, 1KG, and HGDP, we systematically investigated and evaluated whether the genetic diversity coverage in 1KG was sufficient to represent the high population diversity in Asia, especially in Southeast Asia considering the limited number of population samples included in 1KG. We developed a method to measure and evaluate the coverage of 1KG samples at a scale of Asia-Pacific region based on principal component analysis (PCA) of genome-wide SNP data. Our results have many implications for further study design and provide some directions for international or regional collaborative efforts to investigate human variants.

## MATERIALS AND METHODS

### POPULATIONS AND SAMPLES

We analyzed the latest release of the data (version 3 of phase 1, March 2012 release) of the 1KG and fetched around 36.5M autosomal SNPs of 1,092 individuals representing 14 populations worldwide (The 1000 Genomes Project Consortium, 2010). The genotypes of 938 unrelated individuals representing 53 populations were obtained from the Human Genome Diversity Panel-CEPH (HGDP-CEPH; Rosenberg et al., 2002; Li et al., 2008). The Illumina 650K annotation file with genome version GRCh37 downloaded from UCSC^1^ was used for the coordinates of HGDP dataset to facilitate the combination. For PASNP, we used the Affymetrix GeneChip Human Mapping 50K Xba array annotation file with netaffx-build 32 (GRCh37) from Affymetrix website to call the SNPs from raw CEL files, followed by quality control using the same criteria as used in the original paper (The HUGO Pan-Asian SNP Consortium, 2009). Approximately 57,323 autosomal SNPs were identified from 1,713 individuals representing 71 populations mainly from Southeast Asia and East Asia.

As the variants of individuals from two Asian populations, CDX and KHV, from 1KG had not been released when we initiated this study, we also incorporated the BAM (binary version of Sequence Alignment/Map format) files of 95 individuals from CDX and 49 individuals from KHV downloaded from 1KG website.

### VARIANTS CALLING FOR CDX AND KHV POPULATIONS

The well-curated BAM files from CDX (95) and KHV (49) were processed using the GATK (McKenna et al., 2010; GATK-1.2-2-g8143) to call the raw variants, followed by variant quality recalibration (Depristo et al., 2011) of the default pipeline of next generation sequence data SNP calling at the Broad Institute. Around 16.9M SNPs were identified by joint calling across all samples from CDX and KHV. Then the SNP loci with QUAL value no less than 100 and individual SNP loci with DP (single locus sequencing depth) no less than 4 were applied to keep the high-quality SNPs for each individual. Then the individuals with missing data larger than 30%, or loci with missing genotypes larger than 20% in any of the three databases (1KG, HGDP, and PASNP) were excluded. The remaining 18,874 SNPs of 60 individuals from CDX and 43 from KHV were used for the data combination.

### COMBINED GENOTYPE DATA

As the datasets were from sequencing or genotyping on different platforms, we identified a set of intersected SNPs among these three datasets and used only data from autosomal chromosomes. Any SNP with discordant annotation among any pair of populations is not included in the combined genotype data, for example, strand bias (SNPs with A/G in PASNP and C/G in 1KG, or SNPs with A/T in HGDP but A/C in 1KG) and annotation misleading (rs1611684 of chr6:29825846 in HGDP is A/C, but the individual genotype is AA, AG, or GG). Next, the SNPs with missing genotypes larger than 20% in a population were discarded. Finally, we identified a set of 7,775 clean autosomal SNPs from 3,832 individuals in these three datasets. We noted that the number of SNPs was smaller than the maximum set of overlapping SNPs shared among these datasets, as some SNPs were filtered out during the quality control procedures.

### GEOGRAPHIC LOCATION CLASSIFICATION

**FIGURE 1:**
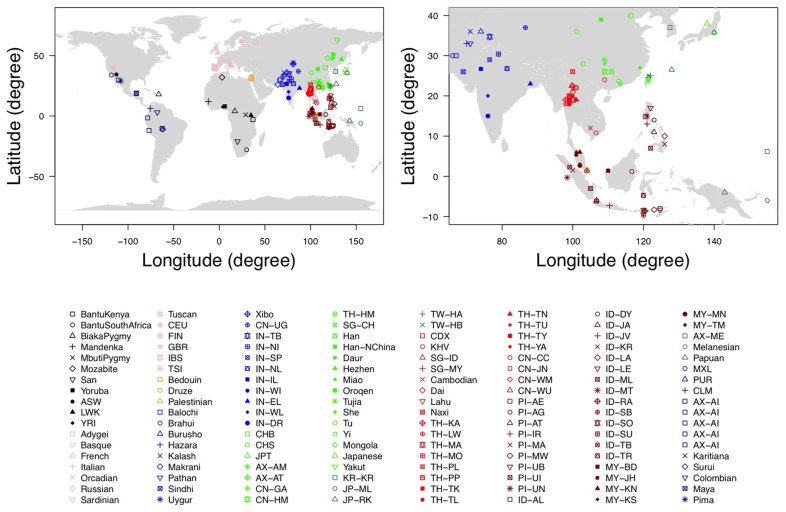
**The geographic location of each population.** The figure on the left is the documented geographic locations of all the 140 populations used in our analysis, while the figure on the right is the magnified version in East Asians and Southeast Asians.

We divided the original geographic location of each population in the combined data into eight main areas, i.e., Africa, Native America, Europe, West Asia, Central and South Asia, East Asia, Southeast Asia, and Oceania (Table 1). The original geographic location of each population was obtained from the publication of each dataset (**Figure 1**). Considering the great influence of population admixture and complex human migration history in Southeast Asia as well as the substantial linguistic diversity, we used the linguistic affiliations mentioned in the original publication (The HUGO Pan-Asian SNP Consortium, 2009) to facilitate the classification. For example, the TH-HM population living in Thailand is of Hmong-Mien, Hmongic, Chuanqiandian linguistic affiliation, which has the same linguistic affiliation as that of CN-HM population from Guizhou, China. Therefore, the TH-HM population was considered as an East Asian population. Due to the fact that Yunnan in China is adjacent to Southeast Asia and the possible migration among these regions, CDX and KHV populations are considered as Southeast Asian populations. To compare the population genetic diversity difference between Southeast Asia and East Asia, we classified the populations in PASNP into nine linguistic groups as mentioned in the original paper, i.e., Altaic (JP-ML, JP-RK, KR-KR, CN-UG), Sino-Tibetan (SG-CH, TH-KA, TW-HA, TW-HB, CN-GA, CN-JN, IN-TB), Hmong-Mien (TH-HM, TH-YA, CN-HM), Tai-Kadai (TH-TK, TH-TL, TH-TU, TH-TY, CN-CC), Austro-Asiatic (MY-JH, MY-KS, TH-LW, TH-MA, TH-MO, TH-PL, TH-PP, TH-TN, CN-WM, CN-WU), Papuan (AX-ME), Dravidian (SG-ID, IN-DR), Indo-European (IN-EL, IN-IL, IN-NI, IN-NL, IN-SP, IN-WI, IN-WL), with the rest populations all belonging to Austronesian.

**Table 1:** The population identities from 1KG, HGDP, and PASNP for each one of the eight main areas.

Region	1KG	PASNP	HGDP
Africa	YRI, LWK, ASW		Bantu Kenya, Bantu South Africa, Mandenka, Yoruba, San, MbutiPygmy, Biaka, Mozabite
Native America	MXL, PUR, CLM	AX-AI	Karitiana, Surui, Colombian, Maya, Pima
Europe	CEU, TSI, GBR, FIN, IBS		Orcadian, Adygei, Russian, Basque, French, Italian, Sardinian, Tuscan
West Asia			Bedouin, Druze, Palestinian
Central and South Asia		CN-UG, IN-*, SG-ID	Balochi, Brahui, Makrani, Sindhi, Pathan, Burusho, Hazara, Uyghur, Kalash, Xibo
East Asia	CHB, CHS, JPT	KR-KR, JP-ML, JP-RK, TW-HA, TW-HB, AX-AT, AX-AM, CN-GA, CN-HM, TH-HM, SG-CH	Han-N China, Han, Daur, Hezhen, Miao, Oroqen, She, Tu, Yi, Mongolia, Japanese, Yakut
Southeast Asia	CDX, KHV	SG-MY, CN-CC, CN-WA, CN-SH, CN-JN, TH-*, ID-*, PI-*, MY-*	Dai, Lahu, Tujia, Naxi, Cambodian
Oceania		AX-ME	Melanesian, Papuan

Populations from PASNP are denoted according to their locations and the ethnicity except Affymetrix. AX, Affymetrix; CN, China; ID, Indonesia; IN, India; JP, Japan; KR, Korea; MY, Malaysia; PI, Philippine; SG, Singapore; TH, Thailand; TW, Taiwan. For example, CN-UG (Uyghur), SG-ID (Southern India origin), KR-KR (Korean), JP-ML (Japanese), JP-RK (Ryukyuan), TW-HA (Han), TW-HB (Han), AX-AT (Atayal), AX-AM (Ami), CN-GA (Han), CN-HM (Hmong), TH-HM (Hmong), SG-CH (Chinese), SG-MY (Malay), CN-CC (Zhuang), CN-WA (Wa), CN-SH (Han), CN-JN (Jinuo), and AX-ME (Melanesian). For the purpose of presentation, a group of populations from each country are compressed as follows. IN-* includes IN-TB (Ladakhi Buddhist), IN-NI (Tharu, Himalayan Tribe), IN-SP (Upper caste, Vashiya), IN-NL (Upper caste, Brahmin), IN-IL (Upper caste, Vashiya), IN-WI (Bhil, Northwest Tribe), IN-EL (Upper caste, Kayastha), IN-WL (Upper caste, Brahmin), IN-DR (Upper caste, Brahmin). TH-* includes TH-PP (Plang), TH-PL (Palong), TH-TK (Tai Kern), TH-TL (Tai Lue), TH-MO (Mon), TH-LW (Lawa), TH-KA (Karen), TH-YA (Yao), TH-TN (H’Tin), TH-MA (Mlabri), TH-TY (Tai Yong), TH-TU (Tai Yuan). ID-* includes ID-AL (Alorese), ID-DY (Dayak), ID-JA (Javanese), ID-JV (Javanese), ID-KR (Batak Karo), ID-LA (Lamaholot), ID-LE (Lembata), ID-ML (Malay), ID-MT (Mentawai), ID-RA (Manggarai), ID-SB (Kambera), ID-SO (Manggarai), ID-SU (Sundanese), ID-TB (Batak), ID-TR (Toraja). PI-* includes PI-UB (Urban), PI-AE (Agta), PI-UN (Urban), PI-IR (Iraya), PI-AT (Ati), PI-AG (Aeta), PI-MW (Mamanwa), PI-MA (Manobo), PI-UI (Urban). MY-* includes MY-KS (Negrito), MY-JH (Negrito), MY-KN (Malay), MY-TM (Proto-Malay), MY-MN (Malay), MY-BD (Bidayuh).

### PRINCIPAL COMPONENT ANALYSIS

Principal component analysis was performed with EIGENSOFT version 3.0 (Price et al., 2006) at the individual level across different geographic scales from worldwide to Asia-pacific region. We used an in-house R^2^ script to facilitate the PCA figure plotting. The proportions of variance explained by each principal component were shown in the PCA plot. The top two principal components PC1 and PC2 were chosen for the population genetic diversity comparison, so that each individual was represented by a point in PCA plot.

### A STATISTIC TO MEASURE GENETIC DIVERSITY COVERAGE

The assumption of our approach was that the genetic diversity of a certain population or region could be measured by the representation of individuals in a two-dimensional space defined by a number of PCs, for instance, PC1 and PC2. To quantitatively evaluate the genetic diversity in Asian populations between two different datasets, such as 1KG and another dataset, we defined *D *_d_ as follows:

(1) $$\begin{array}{c} D_{d\,}\,=\log\frac{\overset{S}{\underset{s=1}{\Sigma }}\delta_{P(K_{1i,}K_{2i})\in R_{s}\left(d_{1},d_{2}\right)}}{\overset{S}{\underset{s=1}{\Sigma }}\delta_{P(O_{1i,}O_{2i})\in R_{s}\left(d_{1},d_{2}\right)}}, \end{array}$$

where *P*(*K *_1__i_ , *K *_2__i_ ) denotes one point (PC1 = *K *_1i_ , PC2 = *K *_2i_ ) in PC plot representing individual *i* from 1KG, while *P*(*O *_1i_ , *O *_2i_ ) represents individual *j* from HGDP and PASNP. The PC plot can be divided into *S* rectangles by a grid with side-length *d*_1_ and *d*_2_. δ _P(K_ 1i_ ,K_2i_ )∈R_s_ (d_1_ ,d_2_ ) was counted once if there is any individual located in the rectangle defined by *R *_s_ (*d*_1_,*d*_2_), otherwise 0 if there is no individual in it. The number of rectangles in the grid overlapping with the points representing the individuals from 1KG, HGDP, and PASNP can be an estimation of the coverage for 1KG, and HGDP and PASNP, respectively. Next we defined the logarithm of the ratio to the base *e* as *D *_d_ . The difficult part of *D *_d_ is how to assign *d*_1_ and *d*_2_ to an optimal estimation of the different coverage for the population pairs, which depends on the average distance $\overset{¯}{d_{1}}\;and\,\overset{¯}{d_{2}}$ of the projection of individuals on PC1 and PC2, respectively. We further introduced a coefficient *m* and defined $d_{1}=m\overset{¯}{d_{1}}\,and\,d_{1}=m\overset{¯}{d_{2},}$ where *m* = 1, 2, 3, … 

## RESULTS

### PRINCIPAL COMPONENT ANALYSIS SHOWING UNDER-REPRESENTATION OF SOUTHEAST ASIAN GENETIC DIVERSITY IN 1KG

**FIGURE 2:**
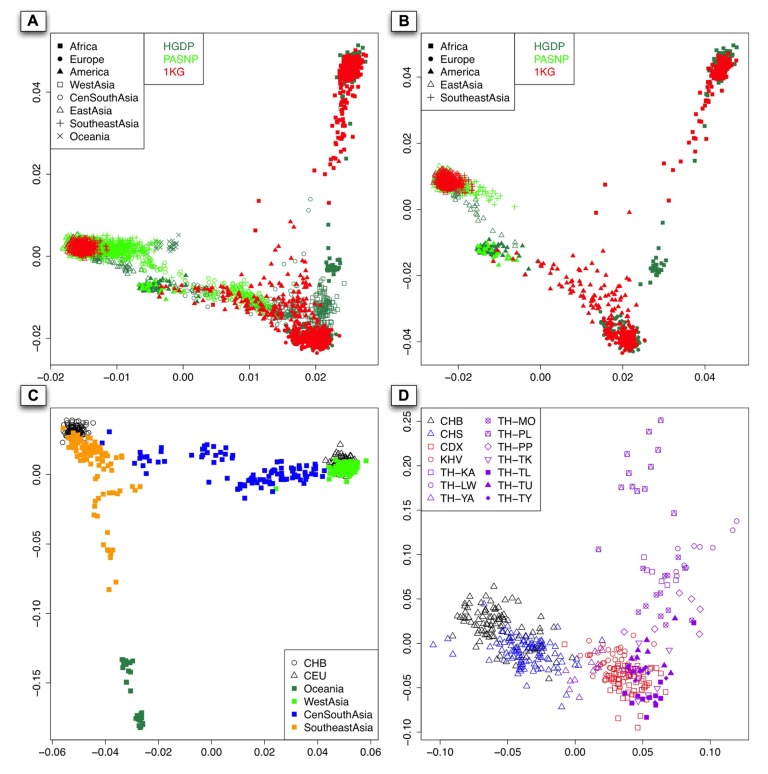
**(A)** The PCA result of all the 140 populations worldwide from 1KG, HGDP, and PASNP without sampling. *x*-Axis denotes the value of PC1, while *y*-axis denotes the value of PC2, with each dot in the figure representing one individual. The color for individuals from 1KG, HGDP, and PASNP are red, sea green, and green, respectively. **(B)** The first two components of PCA result based on randomly selected individuals belonging to Africa, Europe, America, East Asia, and Southeast Asia in the 1KG or HGDP and PASNP have larger sample size in the corresponding geographic area. The colors for each dataset are the same as **(A)**. **(C)**. The PCA result using individuals from CHB, CEU, Oceania, West Asia, Central and South Asia (CenSouthAsia), and Southeast Asia. **(D)**. The PCA result when pooling individuals from CHB, CHS, CDX, KHV, and individuals from Thailand except for TH-MA and TH-TN.

We performed PCA with EIGENSOFT version 3.0 (Price et al., 2006) for all the 3,813 available individuals from 140 populations worldwide, and a two-dimensional plot based on the top two PCs was shown in **Figure 2A**. As expected, individuals from Asia, Europe, and Africa can be well distinguished. The over-representing of East Asian and Southeast Asian samples from HGDP and PASNP than those from 1KG was due to the larger sample size in HGDP and PASNP, so we randomly selected individuals from each geographical area to match sample size so that our results are not biased by the fluctuation of sample size in different regions. As there were no West Asian, Central and South Asian, and Oceanian individuals in 1KG phase I, we removed individuals from those regions in HGDP and PASNP and did PCA again (**Figure 2B**). The result showed that 1KG has lower coverage in East Asia and Southeast Asia compared to HGDP and PASNP, but reasonable coverage in America. For example, 1KG does not have any representation for some Asian populations such as Yakut (empty triangles in sea green) and Pacific islanders (green crosses) from Southeast Asia.

Since Asia is the most populous continent with the largest population size, and PCA also showed considerable population substructure in this area (**Figure 2C**), we examined the diversity representation of 1KG in different parts, i.e., West, Southeast, Central, and South Asia. Since there is no sample or data available for Central and South Asian populations in the latest version of 1KG (phase I), we merged Central Asian and South Asian samples in our analysis. The result also showed substructures in Southeast Asians, for example, Southeast Asian individuals could be roughly classified as those closely related to Han Chinese (CHB) and those showing apparent differentiation from Han Chinese. To examine whether CDX and KHV are genetically closer to Southeast Asian populations than to East Asian populations, we performed PCA of CDX, KHV together with samples from East Asians (CHB and CHS) and Southeast Asians (populations from Thailand). As a result, CDX and KHV were clustered with populations from Southeast Asia instead of those from East Asia. This result confirmed that CDX and KHV should be classified as Southeast Asian (**Figure 2D**). However, as shown below, the coverage of individuals from CDX and KHV indicated insufficient genetic diversity of Southeast Asia in 1KG.

**FIGURE 3:**
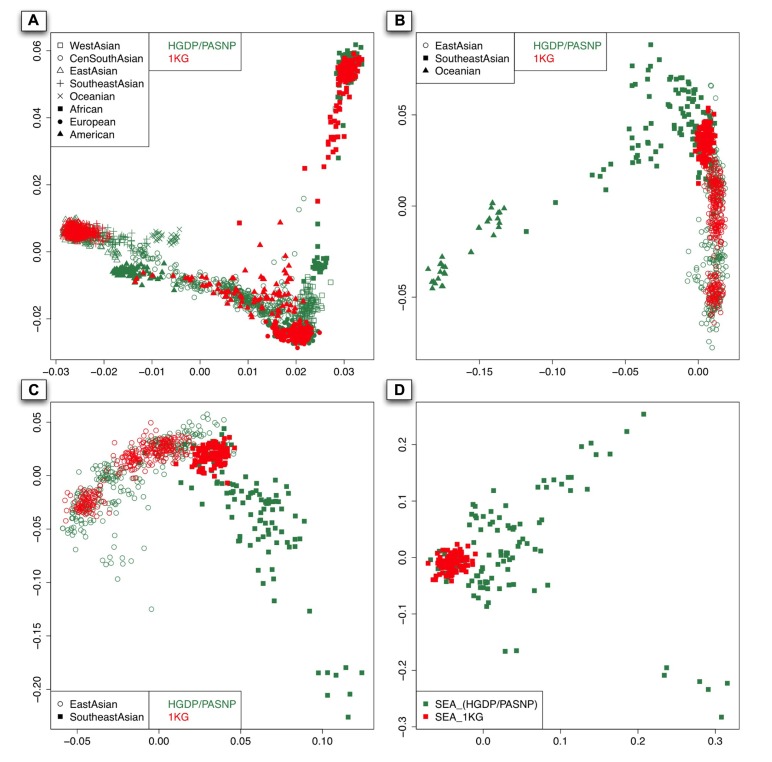
** The first two components of the PCA result with sampling approach based on different contexts: worldwide population context (A)**. East Asian and Pacific islander context **(B)**. East Asian and Southeast Asian contexts **(C)**. and Southeast Asian context **(D)**. The individuals with red color are from 1KG and those with sea green color are from the other two datasets.

As shown in the PC plot based on all 140 populations (**Figure 2A**) displaying population genetic diversity of different geographic areas in a context of worldwide population, it is clear that the area occupied by the individuals representing Southeast Asian and East Asian populations from HGDP and PASNP is larger than that by the individuals from 1KG. When we compared such difference in individuals from Native American populations, we found that the area occupied by the individuals from HGDP and PASNP is smaller than that of the individuals from 1KG. Such results indicated that 1KG had higher genetic diversity coverage in Native American populations but lower genetic diversity coverage in Southeast Asian and East Asian populations. To evaluate such difference of extended genetic diversity measured by the occupied area in a geographic region in an unbiased way, we pooled all 140 populations from eight main geographic areas, and randomly sampled equal number of individuals for each geographic area to eliminate the bias introduced by different sample sizes in 1KG, HGDP and PASNP. In this analysis, we also included samples from Central and South Asia, Oceania, and West Asia. The sample size of populations representing Africa, Native America, Europe, West Asia, Central and South Asia, East Asia, Southeast Asia, and Oceania in 1KG (and in HGDP and PASNP) was 130 (130), 89 (89), 157 (157), 0 (134), 0 (438), 286 (286), 103 (103), and 0 (32), respectively. After this sampling approach based on worldwide population context, we ran PCA again and observed lower genetic diversity coverage of populations representing Southeast Asians and East Asians from 1KG (**Figure 3A**). We were also interested in evaluating whether 1KG has sufficient diversity coverage in different scales besides worldwide samples comparison. We used similar sampling approach as described above and pooled population samples from East Asia, Southeast Asia, and Oceania (**Figure 3B**). We examined distribution of 1KG samples in the PC plots in the context including both Southeast Asian and East Asian populations (**Figure 3C**), and also in the context including only Southeast Asian populations (**Figure 3D**). The 1KG lacks sufficient representation of samples from Southeast Asian populations compared with HGDP and PASNP data in all the four contexts (*p* = 4 × 10^-^^5^, 3 × 10^-^^22^, 3 × 10^-^^24^, and 2 × 10^-^^25^ for each context, respectively, two-dimensional Kolmogorov–Smirnov test). Therefore, the above results indicated that 1KG lacks sufficient coverage of Southeast Asian populations.

To quantitatively evaluate the genetic diversity coverage of 1KG, we developed a statistic *D *_d_ to measure the representation of population samples based on PCA results. To systematically measure such representation in different contexts, we examined eight more groups based on their geographical distributions of populations: non-Africans (NA), Eurasians and Oceanians (EAO), Asians and Oceanians (AO), non-Western Asians and Oceanians (NWAO), East Asians and Pacific islanders (EPI), East Asians and Southeast Asians (ESEA), Southeast Asians and Oceanians (SEAO), and Southeast Asians (SEA). Again, we applied similar sampling procedure to reduce the effect of unbalanced sample sizes between 1KG and the control groups.

**FIGURE 4:**
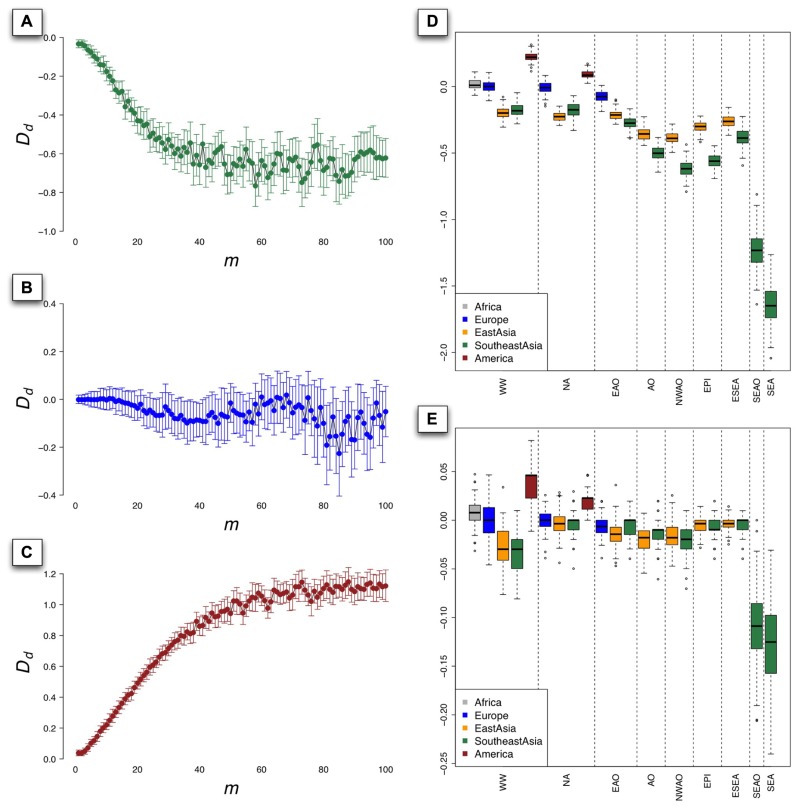
**The value of *D*_d_ as a function of *m* for populations from Southeast Asia (A), Europe (B), and Native America (C) based on 100 PCAs using sampling approach under worldwide populations context, bars indicating the standard deviation (SD) of *D*_d_**. The boxplot of the value of *D*_d_ derived from 1000 PCAs using sampling approach under different population contexts, with *m* = 10 **(D)** and *m* = 1 **(E)**. The nine population contexts are worldwide (WW), non-Africans (NA), Eurasians and Oceanians (EAO), Asians and Oceanians (AO), non-Western Asians and Oceanians (NWAO), East Asians and Pacific islanders (EPI), East Asians and Southeast Asians (ESEA), Southeast Asians and Oceanians (SEAO), and Southeast Asians (SEA).

To investigate the influence of *m*, which can be treated as a coefficient of $\overset{¯}{d_{1}}(d_{1}=m\overset{¯}{d_{1}})\,and\,\overset{¯}{d_{2}}\left(d_{2}=m\overset{¯}{d_{2}}\right)$ used to define the size of rectangle in the grid of the PCs (see Materials and Methods), we set *D *_d_ as a function of *m* and displayed the relationship of *m* and *D *_d_ in **Figure 4**. In the context of worldwide populations, *D *_d_ for Southeast Asians went down steeply while *m* increased from 1 to 20 (**Figure 4A**) and showed considerable fluctuations subsequently beyond 20. To illustrate the performance of *D *_d_ in the situation of good representation of population samples, we evaluated coverage of 1KG in European populations using *D *_d_ based on HGDP and PASNP data. The results showed that *D *_d_ did not deviate significantly from 0 in the context of European populations, even with different values of *m* (**Figure 4B**). Interestingly, the *D *_d_ values have quite different behavior in the context of Native Americans, which were larger than 0 and positively correlated with *m* (**Figure 4C**). This pattern indicated that Native American samples are over-represented in 1KG compared with those in HGDP and PASNP data, which were also supported by the result illustrated by **Figure 2B**. Since our method was based on PCA results and the individuals with similar coordinates in PC plots are likely to be genetically closely related, we chose only one individual as proxy of diversity in the area defined by *m* to reduce redundancy. We suggested that the value of *m* should be neither too small nor too large. Especially, *m* = 1 does not make any sense since in this case any two individuals would not be likely to be in the same rectangle defined by *R*_s_ (*d*_1_,*d*_2_). When *m* was given a very large value, the probability of two distantly related individuals from different populations to be located in the same rectangle would be very high. In both cases, it would lead to unreasonable estimations.

To evaluate genetic diversity coverage of 1KG in a specific geographic area, we calculated *D *_d_ with the sampling procedure repeated for 100 times using *m* = 10. The results of *D *_d_ for Native American populations based on worldwide population context (WW) and NA context were all significantly greater than 0 (*p* = 2 × 10^-^^84^ and 4 × 10^-^^50^, Student’s *t*-test; **Figure 4D**), which indicated high genetic diversity coverage of 1KG in this area. The values of *D *_d_ for European populations under WW context and NA context did not significantly deviate from 0 (*p* = 0.66 and 0.13), but less than 0 based on EAO context (*p* = 5.2 × 10^-^^32^). However, the values of *D *_d_ for East Asian populations were all significantly less than 0 (*p* < 7 × 10^-^^64^), even if we set *m* = 1 in the calculation (*p* = 0.006 for NA and *p* < 10^-^^6^ for other contexts; **Figure 4E**). The results for Southeast Asian populations based on AO, NWAO, EPI still had lower values of *D *_d_ than those for East Asian populations, which indicated that the genetic diversity coverage of 1KG in Southeast Asia was much less sufficient. As we looked closer, the only two population samples (KHV and CDX) collected from Southeast Asia in 1KG could represent a small proportion of genetic diversity of this region, as can also be visualized from PCA result (**Figure 3D**). Therefore, the coverage of population genetic diversity in 1KG is particularly insufficient in Southeast Asia.

## DISCUSSION

In this study, we developed a method to quantitatively evaluate the genetic diversity coverage of 1KG based on PCA of genome-wide SNP data in 140 populations worldwide. Our results showed insufficient representation of genetic diversity of some geographic regions in 1KG, especially of Southeast Asia. Although our analysis showed that 1KG seemingly has reasonable coverage in many regions such as Africa, Europe and East Asia, we would like to point out that our results as well as conclusions might be conservative for the following reasons.

Firstly, our analysis relied on comparison of 1KG data and other available reference data such as HGDP and PASNP, which means that we could not give a good evaluation of 1KG data for their representation of genetic diversity if there were not sufficient data available in either 1KG or reference data. For example, there is no Central Asian data available in 1KG while there are data available in reference data from some Central Asian populations such as the Uyghur in both HGDP and PASNP. However, we did not draw a conclusion that 1KG lacks coverage of genetic diversity in Central Asia. Because previous studies reported that Central Asian populations such as the Uyghur are admixed populations with both Caucasian and East Asian ancestries (Xu and Jin, 2008; Xu et al., 2008, 2009), it is not clear whether the genetic diversity of Central Asian populations has already been represented jointly by European and East Asian populations since this relationship could not be revealed by PCA-based analysis. Regarding South Asia, the situation is similar. Since the phase II of 1KG has not yet released the data containing South Asian populations, we could not evaluate the genetic diversity coverage of 1KG data in South Asia in current study.

Secondly, our method was developed based on PCA results. On the one hand, population structure revealed by PCA is sensitive to rare variants but not common variants. We compared the minor allele frequency (MAF) spectrum of our data set and the MAF spectra of the data sets with similar number of random SNPs we created from 1KG data. As a result, our data set did not have excess of rare SNPs compared with the other randomly sampled data sets. On the other hand, PCA can only capture the majority of information in genome-wide data and populations, and fine-scale structures could not be fully revealed with limited dimensions of principal components, although the *D *_d_ statistic can be easily extended to handle three-dimension or even higher dimensions PCs. Therefore, our analysis only evaluated whether 1KG data cover the majority of genetic diversity in a particular geographical region. From this aspect, our results may be conservative, i.e., genetic diversity coverage of 1KG data in those regions showing good representation could be overestimated.

Finally, we noted in our analysis that the sequence error using NGS existed in 1KG, which could affect the estimation of population genetic diversity. Importantly, sequence errors tend to result in a higher estimation of genetic diversity. The consequence, as we discussed above, is overestimating the diversity coverage of 1KG data in those regions with genotyping data being compared. Therefore, regarding this issue, our results as well as conclusion could be conservative.

In summary, we concluded that the current version of 1KG data does not have sufficient genetic diversity coverage in Asia, especially in Southeast Asia that harbors significant cultural and linguistic diversity as well as the majority of genetic diversity in Asia. We thus suggest a good coverage of Asian populations, especially of Southeast Asian populations, be considered in 1KG or a regional effort be initialized to provide a more comprehensive characterization of the human genetic diversity in Asia, which is important for both evolutionary and medical studies in the future.

## Conflict of Interest Statement

The authors declare that the research was conducted in the absence of any commercial or financial relationships that could be construed as a potential conflict of interest.

## Footnotes

1 http://hgdownload.cse.ucsc.edu/goldenPath/hg19/database/

2 http://www.r-project.org
